# Antibacterial properties of tualang honey and its effect in burn wound management: a comparative study

**DOI:** 10.1186/1472-6882-10-31

**Published:** 2010-06-24

**Authors:** Nur-Azida Mohd Nasir, Ahmad Sukari Halim, Kirnpal-Kaur Banga Singh, Ananda Aravazhi Dorai, Mehru-Nisha Muhammad Haneef

**Affiliations:** 1 Reconstructive Sciences Unit, School of Medical Sciences, Universiti Sains Malaysia, Health Campus 16150 Kubang Kerian, Kelantan, Malaysia; 2 Department of Medical Microbiology & Parasitology, School of Medical Sciences, Universiti Sains Malaysia, Health Campus 16150 Kubang Kerian, Kelantan, Malaysia

## Abstract

**Background:**

The use of honey as a natural product of *Apis *spp. for burn treatment has been widely applied for centuries. Tualang honey has been reported to have antibacterial properties against various microorganisms, including those from burn-related diagnoses, and is cheaper and easier to be absorbed by Aquacel dressing. The aim of this study is to evaluate the potential antibacterial properties of tualang honey dressing and to determine its effectiveness as a partial thickness burn wound dressing.

**Methods:**

In order to quantitate the bioburden of the swabs, pour plates were performed to obtain the colony count (CFU/ml). Swabs obtained from burn wounds were streaked on blood agar and MacConkey agar for bacterial isolation and identification. Later, antibacterial activity of Aquacel-tualang honey, Aquacel-Manuka honey, Aquacel-Ag and Aquacel- plain dressings against bacteria isolated from patients were tested (*in-vitro*) to see the effectiveness of those dressings by zone of inhibition assays.

**Results:**

Seven organisms were isolated. Four types of Gram-negative bacteria, namely *Enterobacter cloacae*, *Klebsiella pneumoniae*, *Pseudomonas *spp. and *Acinetobacter *spp., and three Gram-positive bacteria, namely *Staphylococcus aureus*, coagulase-negative *Staphylococcus aureus *(CONS) and *Streptococcus *spp., were isolated. Total bacterial count decreased on day 6 and onwards. In the *in-vitro *antibacterial study, Aquacel-Ag and Aquacel-Manuka honey dressings gave better zone of inhibition for Gram positive bacteria compared to Aquacel-Tualang honey dressing. However, comparable results were obtained against Gram negative bacteria tested with Aquacel-Manuka honey and Aquacel-Tualang honey dressing.

**Conclusions:**

Tualang honey has a bactericidal as well as bacteriostatic effect. It is useful as a dressing, as it is easier to apply and is less sticky compared to Manuka honey. However, for Gram positive bacteria, tualang honey is not as effective as usual care products such as silver-based dressing or medical grade honey dressing.

## Background

The uses of honey as a burn injury treatment were first documented by the Egyptians in 2000 BC [1]. It is well documented that honey inhibits a broad spectrum of bacterial species [2-4]. Also letter to editor from Majtan and Majtan 2009 (personal communication) support this finding.

Honey, which is high in carbohydrates, is produced from bee floral nectar, with fructose and glucose as major components and other honeybee derived chemical compounds or phytochemicals present in small quantities. These combinations and a number of other factors, such as low water activity, hydrogen peroxide, low pH, and other partially characterized or uncharacterized compounds, produce a high antimicrobial action [5-9].

The increasing rate of multi-resistant to parenteral antibiotic and ineffectiveness of antibacterial topical dressings such as Silver sulfadiazine (SSD) which is widely used to represent a major challenge in burn care. This problem required alternative treatment option.

Conventional dressings such as gauze, Tulle gras and hydrofibre based do not address the issues of infection effectively. Therefore new generation of silver based dressing such as Aquacel-Ag^® ^and Acticoat^® ^has been developed to reduce local infection and is related morbidity. At the same time other non conventional treatment approach such as the use of honey has become an attractive alternative[10-12]. Manuka honey is not widely used for the management of burn compared to silver based dressings. The silver based dressing is presently used in most clinical settings with good scientific evidence. Tualang honey has been reported to have antibacterial properties against various microorganisms, including those from burn-related diagnoses such as Pseudomonas spp. and MRSA [2,9,13-15], and it is cheaper and easier to be absorbed by Aquacel dressing. Tualang honey was obtained from the tualang tree (*Koompassia excelsa*) and used as a partial thickness burn treatment.

This partial thickness burn usually extends through the epidermis downward into the papillary, or superficial, layer of the dermis or it can be extended downward into the reticular, or deeper, layer or the dermis [16].

This study evaluated the potential antibacterial properties of tualang honey dressing and its effectiveness compared to Aquacel-Ag dressing to treat partial thickness burn wounds patient. Tualang honey was chosen as it is a Malaysian honey used to treat local people. It has been reported from various parts of the world that various types of honey differ substantially in their activity, depending on the nectar source [6]. Later, in this study, Aquacel-Ag dressings, Aquacel-Tualang, Aquacel-plain (as a control) were used in *in-vitro *antibacterial studies to make it comparable to our clinical treatment. Bacteria isolated from patients with partial thickness burn wounds were treated with all of the above dressings to assess the dressing's antibacterial effect.

## Methods

### Dressing samples

Tualang honey was supplied by the Federal Agriculture Marketing Authority (FAMA) office in Kedah, Malaysia. The tualang honey was sterilized by gamma radiation (25 kGy) for clinical use, and commercial manuka honey (Kordel's UMF10+) was used for laboratory use. Aquacel^® ^(plain dressing) and Aquacel^® ^Ag were purchased from ConvaTec, E.R. Squibb & Sons, L.L.C, US. for clinical and laboratory use. The study aim was to compare Aquacel-tualang honey (Aquacel plain soaked with tualang honey) with Aquacel^® ^Ag (as it was known as a standard dressing in clinical practice) to see its potential antibacterial effect. Whereas, manuka honey was well known having antibacterial effect and used as a dressing. Tualang honey is easier to be absorbed by Aquacel plain compared to Manuka honey. Furthermore tualang honey is easy to access as it is Malaysian honey and cheaper compare to manuka or Aquacel-Ag dressing.

### Selection of Patients and Treatment Procedure

This study has been approved by Universiti Sains Malaysia Human Ethical Committee. A total of 10 patients, ages 2 - 60 years old, with partial thickness burns (< 30%) and requiring tangential excision were included in this study after consent. Subjects were expected to survive and were kept in a hospital environment. Selected patients were screened on day 0 to day 2. Dressings were applied on day 3 (randomly treated with either Aquacel-Tualang honey dressing or Aquacel-Ag dressings). Dressing was on day 6 and day 9 (only if patient delayed for surgery). Swabs were taken at every dressing change.

### Surface Swabs

Two surface swabs from 2-5 cm^2 ^of burn wound were collected from each patient on day 0 before applying any dressing material. One swab was put in normal saline and the other one was kept in a container without normal saline. The collected samples were immediately processed for bacteriological analysis. Later, swabs were collected on day 3, day 6, day 6+ or on the day of surgery.

### Bacteria identification

Swabs collected were streaked on blood agar and MacConkey agar plates for bacterial identification. The plates were incubated at 35 ± 2°C for 24 hours. Further identification on the microorganisms' growth on the plates (if any) was done using the conventional method or using the RapID™ STR or RapID™ SS/u System (Remel Inc. USA).

### Total bacterial count determination

The other swab obtained from the same patient was immersed in 2 ml of normal saline and 10 fold serial dilutions were performed. Then, 1 ml of each dilution was pipetted onto petri dishes in triplicate. All the plates were incubated at 35 ± 2°C for 24 hours. Colony counts were performed on each plate and were recorded as CFU/ml.

### Measurement of zone of inhibition

Bactericidal activity was tested against each bacteria identified from the burn wound. This was done to compare the zone of inhibition obtained when the bacteria were treated with Aquacel-Tualang honey dressing, Aquacel-Manuka honey dressing Aquacel-Ag dressing and Aquacel-plain dressing (as a control) by zone of inhibition assay [17]. The Aquacel plain dressings were cut into 1 cm × 1 cm squares each and were soaked with 200 μl of pure Tualang honey or 200 μl of pure Manuka honey. Inocula of 0.5 MacFarland (1 × 10^8 ^CFU/ml) microorganisms were prepared and inoculated onto a Mueller-Hinton agar (MHA) plate. Inoculation was done within 15 minutes of preparation time. Then, the four different dressings were placed onto the MHA plate. The test was done in triplicate. All plates were incubated overnight at 35 ± 2°C, and the zone of inhibition was measured after 24 hours of incubation.

## Results

### Identification

Table 1 shows the 7 different bacteria that were isolated and identified from 10 patients. Four types of Gram-negative bacteria, namely *Enterobacter cloacae*, *Klebsiella pneumoniae*, *Pseudomonas *spp. and *Acinetobacter *spp., and three Gram-positive bacteria, namely *Staphylococcus aureus*, coagulase-negative *Staphylococcus aureus *(CONS) and *Streptococcus *spp., were isolated. *S. aureus *was isolated in most of the swab samples having mixed growth.

**Table 1:** Bacteria isolated and colony count from burn wounds treated with tualang honey and silver-based dressing

Sample	Treatment	Days	Microorganisms	CFU/ml
1	Aquacel plain	0	NG	
	Aquacel-tualang honey	3	*E. cloacae*	
		6	*E. cloacae*	0.97×10^1^
		6+	NS	
2	Aquacel plain	0	NG	
	Aquacel-tualang honey	3	*S. aureus *&*Streptococcus *spp.	3.3 × 10^3^
		6	*Streptococcus *spp.	3.3 × 10^3^
		6+	*S. aureus *& *Streptococcus *spp.	2.4 × 10^5^
3	Aquacel plain	0	NG	
	Aquacel-tualang honey	3	*K. pneumoniae *& *S. aureus*	5.3×10^4^
		6	NS	
		6+	NS	
4	Aquacel plain	0	NG	
	Aquacel-tualang honey	3	*S. aureus *& CONS	2.0 × 10^1^
		6	NS	
		6+		
5	Aquacel plain	0	NG	
	Aquacel-tualang honey	3	CONS	1.6×10^2^
		6	*K. pneumoniae*	5.9×10^5^
		6+	CONS, *K. pneumoniae *& *Pseudomonas *spp.	9.3×10^3^
6		0	CONS &*S. aureus*	5.9×10^1^
	Silver	3	CONS &*S. aureus*	2.3×10^2^
		6	NG	
		6+	NS	
7		0	NG	
	Silver	3	NG	
		6	NS	
		6+	NS	
8	Aquacel plain	0	NG	
	Aquacel-tualang honey	3	NG	
		6	NS	
		6+	NS	
9	Aquacel plain	0	NG	
	Aquacel-tualang honey	3	CONS & *Acinetobacter *spp.	2.0 × 10^3^
		6	NS	
		6+	NS	
10	Aquacel plain	0	NG	
	Aquacel-tualang honey	3	*Pseudomonas *spp. *Acinetobacter *spp.	3.8 × 10^2^
		6	*Pseudomonas *spp. *Acinetobacter *spp.	6.8 × 10^5^
		6+	NS	

CONS - coagulase-negative *S. aureus*, NG - no growth, NS - no sample

On day 0 (admission day), swabs were taken on the partial thickness burn wound patients. Days 0 - 2 were a screening period which is only Aquacel plain was given to the patients. Only sample 6 yielded a positive culture, with mixed growth of CONS and *S. aureus*; the other nine yielded no bacterial growth.

On day 3, 2 single cultures from samples 1 (*E. cloacae*) and 5 (CONS) were obtained, and 6 yielded mixed bacterial growth. Those mixed growths contained two microorganisms.

Day 6 showed microorganisms isolated from 5 samples of burn wounds 3 days after treatment. Samples 1, 2 and 5 yielded single growth of *E. cloacae*, *Streptococcus *spp. and *K. pneumoniae*, respectively, while sample 10 yielded mixed growth of *Pseudomonas *spp. and *Acinetobacter *spp. Sample 6 yielded no growth.

Sampling after day 6 was less, as patients went for early surgery as soon as their wound was stabilized. Mixed bacterial growth was observed in both samples collected after day 6 when treated with honey. Two bacteria were isolated from sample 2 (*S. aureus *and *Streptococcus *spp.), whereas three types of bacteria were isolated from sample 5 (CONS, *K. pneumoniae *and *Pseudomonas *spp.)

### Total bacterial count

Table 2 shows that burn wounds treated with the silver-based dressing yielded less than 10^4 ^CFU/ml for sample 6, while sample 7 showed no microorganisms isolated.

**Table 2:** Total bacterial count of swab samples treated with Aquacel-tualang honey dressing and Aquacel-Ag

Sample	Treatment	CFU/ml			
		
		Day 0	Day 3	Day 6	> Day 6
1	Aquacel-tualang honey	NG	NG	< 10^4^	NS
2	Aquacel-tualang honey	NG	< 10^4^	< 10^4^	≥10^4^
3	Aquacel-tualang honey	NG	≥10^4^	NS	NS
4	Aquacel-tualang honey	NG	< 10^4^	NS	NS
5	Aquacel-tualang honey	NG	< 10^4^	≥10^4^	< 10^4^
6	Aquacel-Ag	< 10^4^	< 10^4^	NG	NS
7	Aquacel-Ag	NG	NG	NS	NS
8	Aquacel-tualang honey	NG	NG	NS	NS
9	Aquacel-tualang honey	NG	< 10^4^	NS	NS
10	Aquacel-tualang honey	NG	< 10^4^	≥10^4^	NS

NG - No growth, NS - No sample

Burn wounds treated with tualang honey showed an increase in CFU/ml for sample 3 on day 3. The CFU/ml was ≥10^4 ^on day 6 for samples 5 and 10, and on day 6 onward, sample 2 showed a CFU/ml ≥10^4^.

In this study, bioburdens of the swab were determined in each patient on days 0, 3, 6 and > 6. On day 0, no bacteria was isolated from 90% of the samples, and only 10% yielded growth of bacteria. On day 3, 70% of the samples showed growth of microorganisms, in which only 10% of samples gave ≥10^4 ^CFU/ml. On day 6, 40% of swabs showed growth of bacteria, in which 20% were ≥10^4^, and no growth was observed in 10% of the samples tested. On day 6, 50% of the swab samples could not be collected. Only 2 swab samples were obtained after day 6, which resulted in < 10^4 ^CFU/ml and ≥10^4 ^CFU/ml, respectively.

### Zone of inhibition

Table 3 shows that all dressings had antibacterial effects against all the microorganisms isolated from burn wounds by an *in-vitro *assay. The silver-based dressing (Aquacel-Ag) showed better antibacterial effects than other dressings. Aquacel-Ag dressing was the most effective antimicrobial agent against Gram-negative bacteria, and it was most effective against *Pseudomonas *spp., with a zone of inhibition 18 mm. However, Aquacel-Ag and Aquacel-Manuka honey dressings gave better zone of inhibition for Gram positive bacteria compared to Aquacel-Tualang honey dressing. The highest zone of inhibition was obtained for streptococci treated with Aquacel-Manuka honey dressings. The zone of inhibition obtained for Gram negative bacteria were comparable when treated with Aquacel-Manuka honey and Aquacel-Tualang honey dressing.

**Table 3:** Antibacterial effect of selected dressings on isolated bacteria from burn wounds

Bacteria strains	Average zone of inhibition (mm)			
	
	Aquacel- Tualang honey (100%)	Aquacel-Manuka honey (100%)	Aquacel-Ag	Aquacel (Plain)
*Staphylococcus aureus*	11	15.3	13.2	0
*Coagulase-negative Staphylococcus aureus*	11	14.7	14.3	0
*Streptococcus *spp.	12.8	16.8	13.7	0
*Enterobacter cloacae*	10.5	11.2	12.3	0
*Klebsiella pneumoniae*	10.5	11.2	12.3	0
*Pseudomonas *spp.	11	11.8	18	0
*Acinetobacter *spp.	11	12.3	13.5	0

## Discussion

This study used Aquacel-tualang honey dressing and Aquacel-Ag dressing to treat partial thickness burn patients clinically and to determine their antimicrobial effects *in vitro*. Aquacel plain was soaked with tualang honey and used as a dressing instead of being taken orally (systemic agent) [18]. The superficial swab was chosen to collect samples, in order to identify the commonly isolated microorganisms from the burn wound. This method was simple, inexpensive and less painful [19]. A previous study had shown no significant differences in bacterial counts between full thickness and partial thickness burns, whether by surface swab or by biopsy [20].

No infection or bacteria were isolated on day 0 (first day of injuries), as the burn wound surface is sterile immediately following thermal injury, as reported by Church and colleagues [21]. Gosh and colleagues [22] reported that re-epithelialization of the wound begins within hours after injury by the movement of epithelial cells from the surrounding epidermis over the denuded surface. Rapid reestablishment of the epidermal surface and its permeability barrier prevents excessive water loss and time of exposure to bacterial infections, which decreases the morbidity and mortality of patients who have lost a substantial amount of skin surface. Unfortunately, as thermal injuries impair the skin and its normal barrier function, contamination and infection is almost unavoidable, even with the use of topical antimicrobial agents [23]. In this study, bacteria were isolated on day 3, as the colonization and infection began [14,21]. Infection of the burn wound is indicated if the number of bacteria is ≥10^4 ^CFU/ml or mg [21,24]. The infection rate increased until day 6 and more (> day 6) if the patient was delayed for surgery. Otherwise, no swabs or tissues were obtained after day 6, as surgery was done as soon as the patient was hemodynamically stabilized [13,21]. The early burn wound excision that now occurs within the first few days after burn injury has resulted in improved survival rates. The primary aims of early excision are removal of the dead tissue that stimulates an overwhelming systemic inflammatory response syndrome and prevention of infection by temporary or permanent closure of burn wound [21].

The most common microorganism identified in this study was *S. aureus *followed by CONS, *K. pneumoniae*, *Streptococcus *spp., *E. cloacae*, *Pseudomonas *spp. and *Acinetobacter *spp. These microorganisms were commonly isolated from partial thickness burn wounds. Similar results were reported by Dastouri and colleagues [25], Basualdo and colleagues [14] and Pieper [9]. These microorganisms showed differences in zones of inhibition when treated with Aquacel plain dressing soaked with pure tualang honey, Aquacel plain dressing soaked with pure manuka honey and Aquacel-Ag dressing, with Aquacel plain as the control.

The disc diffusion assay showed that Aquacel-manuka honey dressing had almost the same antibacterial effect as Aquacel-Ag dressing and was more effective than Aquacel-tualang honey dressing against Gram-positive bacteria, such as *S. aureus*, CONS and *Streptococcus *spp.

The Aquacel-Ag dressing was the most effective dressing against the four Gram-negative bacteria isolated from burn wounds [10] Aquacel-manuka honey dressing gave slightly higher MIC values compared to Aquacel-tualang honey dressing for all the bacteria tested. Plain Aquacel dressing was used as the control in this study, and it had no antibacterial effect against any of the microorganisms tested.

Aquacel-tualang honey worked more effectively if the dressing was applied and covered the wound area properly. Gentle pressure applied to the dressing covering the wound area would be very helpful, as reported by Jones and colleagues [26].

Visual inspection of the disc diffusion assay also showed that tualang honey had more partial inhibition (PI)/a lower concentration that retarded growth [15] than manuka honey. This is because tualang honey is more liquid than manuka honey and can be easily absorbed by MHA and other substances, such as human skin. It was observed in this study that tualang honey took less than one minute to be absorbed through the dressing, whereas manuka honey took almost one hour to be absorbed in the dressing.

## Conclusions

In this study, tualang honey demonstrated to poses substantial bactericidal as well as bacteriostatic effect. The Aquacel dressing soaked with pure tualang honey without any dilution was less sticky than manuka honey and was easier to apply as a dressing. However, for Gram positive bacteria, tualang honey is not as effective as usual care products such as silver-based dressing or medical grade honey dressing.

## Competing interests

The authors declare that they have no competing interests.

## Authors' contributions

NAMN carried out the microbiological analysis and prepared the manuscript, HAS designed the study and drafted manuscript, KKBS involved in microbiological analysis, design of the study and manuscript correction, DAA participated in design of the study and manuscript correction and MNMH involved in microbiological analysis. All authors have read and approved the final manuscript.

## Pre-publication history

The pre-publication history for this paper can be accessed here:

http://www.biomedcentral.com/1472-6882/10/31/prepub
